# Qualitative focus groups with stakeholders identify new potential outcomes related to vaccination communication

**DOI:** 10.1371/journal.pone.0201145

**Published:** 2018-08-01

**Authors:** Jessica Kaufman, Rebecca Ryan, Sophie Hill

**Affiliations:** Centre for Health Communication and Participation, School of Psychology and Public Health, La Trobe University, Melbourne, Victoria, Australia; University of New South Wales, AUSTRALIA

## Abstract

**Introduction:**

Communication interventions are widely used to promote childhood vaccination and sustain vaccine acceptance, but communication’s role in changing people’s beliefs and behaviours is not well understood. To determine why these interventions work or where they fail, evaluations must measure a range of outcomes in addition to vaccination uptake. As part of a larger project to develop a preliminary Core Outcome Set for vaccination communication, we conducted a qualitative focus group study exploring how parents and health professionals perceive and experience communication encounters and what outcomes are relevant to them.

**Methods:**

Focus group participants included parents and health professionals involved in vaccination communication (healthcare providers, researchers and policymakers). Participants discussed their experiences with communication for childhood vaccination, and what made the communication ‘successful’ or 'unsuccessful.'

Our analysis involved two stages: first, we thematically analysed the discussions, identifying key parent and professional themes. In stage two, we used an interpretive analysis approach to translate the themes and quotes into measurable outcomes. We compared these outcomes with outcomes measured in vaccination communication trials (previously identified and mapped).

**Results:**

We held three focus groups with parents (n = 12) and four with professionals (n = 19). In stage one, we identified six parent themes (primarily related to decision-making) and five professional themes (primarily related to intervention planning, delivery and evaluation).

In stage two, we translated 47 outcomes from parents and 73 from professionals (91 total, de-duplicated). All stakeholders discussed attitudes or beliefs and decision-making outcomes most frequently. Most (66%) of the focus group-generated outcomes were not measured in vaccination communication trials.

**Conclusion:**

Consulting with stakeholders through focus groups allowed us to explore how parents and professionals experienced vaccination communication, identify those aspects of the experience that were important to them, and translate these into outcomes that can be prioritised into a Core Outcome Set and measured in intervention evaluations.

## Introduction

Understanding the role of communication in changing people’s health-related beliefs and behaviours is critical to public health, especially with regard to communication about childhood vaccination. Vaccination saves millions of lives every year [1], but many parents have concerns or feel hesitant about vaccinating their children, in some cases delaying or refusing some or all vaccines [2, 3]. Communication with health providers is highly influential in people’s vaccine decision-making–positive communication encounters that build trust can encourage parents to vaccinate [4], while negative experiences can leave parents confused, mistrustful and potentially more hesitant [5]. Vaccination decisions may also be shaped by other types of vaccination communication and messaging, such as websites, government brochures, peer forums and media stories [6, 7].

Research on the effects of different types of communication about childhood vaccination is rapidly increasing, in part because communication and behaviour change are so complex [8–12]. A range of societal, professional and individual factors may shape beliefs and support or hinder vaccination behaviour, and communication interventions can impact or address many of these factors. However, despite the fact that a communication intervention may have multiple aims or components targeting several different behavioural determinants, its effectiveness is frequently assessed only through its impact on the target behaviour, e.g. receipt of vaccination. Failing to measure appropriate intermediate or process outcomes, such as attitudes towards vaccination or satisfaction with the intervention, makes it difficult to understand how or why a communication intervention works, or where it fails [13–15].

To better understand and prioritise the outcomes that should be measured to evaluate the effects of vaccination communication interventions, our broader project aimed to develop a preliminary Core Outcome Set (COS) for these interventions. The development of COS can help improve intervention evaluation by identifying relevant, meaningful and consistent outcomes that should be measured in trials for specific topics or conditions [16, 17]. We began with a literature review to identify and categorise outcomes currently measured in trials (Trial Outcomes Map) [18]. This confirmed that most trials focus on endpoint outcomes like vaccination status, with few considering outcomes related to consumer decision-making or attitudes. In addition to being insufficient to explain why an intervention does or does not work, vaccination endpoint outcomes may not be important to everyone involved in a vaccination communication encounter. For example, parents and professionals do not always share the same view about what communication is seeking to achieve, and what makes a communication encounter ‘successful’ or ‘unsuccessful’. Therefore, to explore people’s views and experiences and identify those outcomes that may be important to them, we conducted a qualitative focus group study.

Qualitative methods are frequently used in COS development to identify additional stakeholder-relevant outcomes [19], which are then added to those identified from trials to form a comprehensive list for prioritisation into the core set [20, 21]. However, to our knowledge, there are no published studies describing this qualitative outcome identification step for preventive health communication or health promotion interventions.

COS research generally focuses on health conditions with physical manifestations or clinical interventions where people can conceptualise potential outcomes relatively easily, but most people do not find it intuitive or straightforward to think about outcomes for a communication intervention. This challenge influenced the way we framed our focus group discussions, as well as our analysis method. Our approach may be beneficial as COS research proliferates across topics and interventions to include those less closely tied to specific clinical or biomedical areas. In this paper, we describe our qualitative focus group study to explore and identify outcomes associated with vaccination communication from a range of stakeholder perspectives.

This study had two aims:

1. To achieve a broad understanding of how parents and professionals (healthcare providers, policymakers and researchers) experience or think about vaccination communication and what makes vaccination communication ‘successful’ or ‘unsuccessful’ to them
2. To link these concepts to commonly-measured outcomes for evaluating vaccination communication interventions and identify outcomes raised by parents and professionals that are not generally measured in trials

The La Trobe University Human Ethics Committee approved this study (ref: S15/148). Permission to recruit participants through advertisements posted in selected Victorian Maternal and Child Health Centres was granted by the Victorian Department of Education and Training (approval 2015_002831).

## Materials and methods

### Study design, recruitment and sampling

We used a qualitative focus group study design. For the purposes of this study, we divided vaccination communication participants into two stakeholder groups: i) parents (i.e. parents or caregivers of young children), and ii) professionals whose work involves vaccination communication.

Parents were eligible to participate if they were involved in or responsible for a decision related to vaccination for a child 6 years old or younger within the previous 12 months. Professionals were eligible for inclusion if they currently worked as:

1. a healthcare provider involved in delivering childhood vaccines or vaccine information (e.g. GP, paediatrician, immunisation or maternal and child health nurse)
2. a researcher involved in vaccination communication or health communication research
3. a policymaker (i.e. a government employee, advocate or employee of a non-governmental organisation (NGO) or multi-national organisation related to childhood vaccination).

We excluded any individuals who were not able to speak and understand English fluently.

We planned to hold two to three focus groups with parents, and at least three with professionals (one with individuals from each of the key professional backgrounds). Empirical evidence about the optimal number of focus groups is inconclusive [22], so our sample size was determined pragmatically, to make the best use of available resources. The researchers perceived no new topics were emerging when data collection ceased. We used a combination of convenience sampling and stratified purposive sampling to recruit participants [23].

Parent participants were recruited using flyers distributed by nurse coordinators in two Maternal and Child Health centres in the Melbourne area, through online advertisements shared via Facebook and other electronic newsletters and message boards, and through snowballing. We recruited professionals purposively to include participants from each stakeholder background. Key informants shared recruitment flyers with their contacts, and international policymaker participants were recruited from a convenience sample of participants attending a related research workshop in Paris (‘Communicate to vaccinate‘ (COMMVAC 2) project expert consultation workshop, Paris, France, September 2015) [6]. All participants received a $30 gift voucher, except the policymakers, whose focus groups were organised during their work day.

### Procedures for focus groups

We screened interested potential participants by phone to ensure that they met the inclusion criteria, and organised focus groups at a time and place that was suitable for the majority of people. We provided refreshments and colouring books and toys to occupy children in the parent sessions. At the start of each session, the lead author explained the nature of the study again and participants signed a consent form. Sessions lasted between 60 and 80 minutes and were audio recorded.

The lead author (JK) facilitated the majority of the sessions. However, participants in one naturally-occurring parent group knew the researcher as well as one another, so a colleague with experience of conducting focus groups acted as facilitator while JK observed.

We used coloured print-outs with icons representing different types of communication interventions and interview guides tailored for parents or professionals to facilitate the discussions (S1 and S2 Appendices). The concept of outcomes can be difficult to explain and discuss with people who do not work and think in a health intervention context, such as parents [20, 24, 25]. This is especially true for a preventive intervention like communication for vaccination, where the outcomes cannot be conceptualised as the resolution or abatement of undesirable symptoms. Therefore, to explore stakeholders’ views on outcomes for vaccination communication, we framed the focus group discussions around the participants’ personal experiences with vaccination communication, and what they felt made the experience or the communication 'good/successful or 'bad/unsuccessful.'

The discussion prompts for parents asked them to describe a recent or memorable vaccination communication experience, how it made them feel, and whether they thought it 'worked' (achieved its aim) or not. They were encouraged to respond to one another's experiences. We then asked them if they had seen or heard about any other forms of vaccination communication, and how they felt about those. We also asked parents an exception question: "What would you like to happen in an ideal communication interaction, and how would you like to feel afterwards?" This is a technique used in family therapy and in other outcomes-related qualitative research [26] to help direct parents to consider what they would like to experience in a hypothetical 'ideal world.'

We asked professionals to describe communication interventions and evaluations they had been involved with or knew about. They described the outcomes that were measured or the informal aims or goals of the communication, and whether it had achieved those goals. They also brainstormed potential outcomes and discussed the relative merits or disadvantages of these.

### Data management and analysis

The audio recordings were professionally transcribed. We coded the transcripts using the software program NVivo 10 [27].

In order both to explore people’s experiences with vaccination communication and link these experiences with measurable outcomes, we conducted a two-stage analysis. In stage one, we performed inductive thematic analysis [28]. JK coded all focus group transcripts using open coding. SH and RR independently read emerging codes and supporting quotations to enhance the accountability of the analysis [29]. Through discussion, the researchers then grouped these inductively-identified codes into a set of parent themes and a set of professional themes related to the participants’ vaccination communication experiences.

While thematic analysis offered insight into the ways the participants think about and experience vaccination communication, it did not produce a list of potentially measurable outcomes that we could prioritise for a COS. Therefore, in stage two of the analysis, we used interpretive analysis to translate the experience-based language of the focus groups into the technical language of academic research. According to Palmberger and Gingrich, “translation transforms insights from the empirical ‘context of discovery’ into the publicized ‘context of academic communication’” [30]. Our analytical approach was informed by interpretive evidence synthesis methods [31], and was shaped by the research team’s knowledge and experience with identifying, defining and classifying communication- and vaccination-related outcomes [18, 32]. We compared the focus group data (themes, specific codes and contributing participant quotes) with the Trial Outcomes Map of all outcomes measured in trials of vaccination communication interventions [18]. Where participants described an experience, feeling or view that corresponded with an existing measurable outcome from the Map, we classified that outcome as having been ‘raised’ in the discussion. Where the data from the focus groups appeared to describe an outcome that could be measured but was not present in the Map, we added this as a new outcome. All translated outcomes were discussed iteratively among the authors. After the translation process, we compared the final list of focus group outcomes with the trial outcomes to identify areas of overlap.

This two-stage analysis process allowed us to capture the purely qualitative experiential data discussed by focus group participants, while also linking these experiences with outcomes that were defined in the language of quantitative evaluation.

## Results

### Participants and sessions

We held a total of seven focus groups: three with parents and four with professionals (there were two policymaker sessions–one Australian and one international) (see Table 1). No participants withdrew. All sessions met face-to-face, except for the professional focus group with healthcare providers, which was convened via teleconference to overcome scheduling conflicts. All data were collected between July 2015 and May 2016.

**Table 1:** Focus group participant details.

STAKEHOLDER GROUP	PARENTS	PROFESSIONALS			
Total number of focus groups	3	4			
Participant type *(number of focus groups)*	Parents *(3)*	Healthcare providers* *(1)*	Researchers *(1)*	Policymakers* *(2)*	
Settings in which the participants were based	Melbourne, Australia	Melbourne and Sydney, Australia	Sydney, Australia	Melbourne, Australia	Cameroon, Mozambique, Nigeria, Switzerland
Total number of participants	12	4	3	5	7
Gender *(n)*	F *(12)* M *(0)*	F *(4)* M *(0)*	F *(1)* M *(2)*	F *(4)* M *(1)*	F *(3)* M *(4)*
Age range *(n)*	18–30 *(0)* 31–45 *(12)* 46–60 *(0)*	18–30 *(0)* 31–45 *(1)* 46–60 *(2)*	18–30 *(1)* 31–45 *(1)* 46–60 *(1)*	18–30 *(0)* 31–45 *(2)* 46–60 *(3)*	18–30 *(0)* 31–45 *(4)* 46–60 *(3)*
Highest level of education *(n)*	High school *(0)* Diploma *(1)* Undergrad *(8)* Postgrad *(3)*	High school *(0)* Diploma *(1)* Undergrad *(2)* Postgrad *(0)*	High school *(0)* Diploma *(0)* Undergrad *(0)* Postgrad *(3)*	High school *(0)* Diploma *(1)* Undergrad *(2)* Postgrad *(2)*	High school *(0)* Diploma *(0)* Undergrad *(0)* Postgrad *(6)*

*One participant did not answer all questions so some data is missing

#### Parents

The three parent focus groups were held in and around Melbourne, Australia and included a total of 12 participants (three, five and four participants per session, respectively). All participants were mothers between 31 and 45 years old, with one or two young children. They were generally highly educated, with the majority holding undergraduate degrees or higher, and participants came from a range of areas in Melbourne. Four were born outside Australia and one spoke a language other than English at home. Of the three parent groups, one was a naturally-occurring group of participants who knew each other through shared membership in a social organisation.

#### Professionals

There were four focus groups with professionals: one each with healthcare providers and researchers, and two with policymakers. The provider session was held via teleconference and included participants from around Australia. The researcher session was held in Sydney. One of the policymaker groups took place in Paris, France and involved international participants. The second policymaker group took place in Melbourne, and involved individuals who worked together.

There were 19 professional participants in total. Some participants held multiple professional roles (e.g. physician researchers, nurse, health department employees). We asked them to comment as much as possible from their perspective as a member of the profession on which their identification and recruitment to focus groups had been based. While recruitment for the healthcare provider focus group targeted both nurses and doctors, all the participants who were ultimately recruited were nurses.

The majority of participants in the professional sessions were female, over the age of 31 and highly educated. The international policymakers were asked where they worked, rather than their countries of origin. Their vaccination work took place in Cameroon, Mozambique, Nigeria, globally or in low and middle income countries more generally.

### Stage 1: Focus group thematic analysis results

#### Parent themes

We identified six themes that describe facets of parents’ experiences with vaccination communication, including their preconceptions, decision-making processes and impressions of the interaction.

THEME 1: CONCERNS OR FEARS (“AN ENDLESS ANXIETY OF EVERY PARENT ABOUT EVERYTHING”). Parents are constantly faced with risks and decisions related to their children's care, and there are a range of different concerns that they weigh up when considering vaccination. Some parents were concerned about the spread of vaccine-preventable diseases: *“I remember being very paranoid because I was travelling on public transport in Melbourne and just being like*, *‘No one cough on me*!*’”*, while other parents were more focused on the potential risks or side effects associated with vaccination: *“[My first child] screamed her face off for like three or four days [after getting vaccinated]…so this time I was terrified that was gonna happen*.*”*

Notably, just because parents had concerns about the safety and potential risks of vaccines didn't necessarily mean they doubted or rejected the need for vaccination, but they did want to discuss these risks: *"I'm going to vaccinate*. *What I want to know is*, *[I want to] have a conversation about those smaller risks* …*and how to mitigate them*.*"*

THEME 2: MAKING A CHOICE. Making the actual decision about whether to vaccinate their children was harder for some parents than for others. One parent said, *"I feel like that needs to be acknowledged*, *that it's not an easy decision being a parent"*, but according to another, *"It hasn't really felt like a decision*, *it's just something I do*.*"* Factors influencing parents’ decision-making included their personal and family background, experience with the health system or experience with vaccine-preventable diseases.

In making their decisions, most parents sought guidance from their healthcare providers, though it wasn’t always helpful: *“I went to [our GP] and I was hoping to get a really definitive answer from him about what to do…I came out of there feeling kind of more confused*.*”* Ultimately, most parents agreed that while they personally believed vaccination was the right choice, everyone should be allowed to weigh the options and decide for themselves: *"If you choose not to vaccinate your child*, *well that's your decision* …*I'm not gonna jump on my high horse and bully a person and call them a bad parent for their decisions*.*"*

THEME 3: CERTAINTY AND CONFIDENCE. All of the parents in the focus groups had vaccinated their children, but they expressed varying degrees of certainty and confidence about this decision. Most were very confident: *"We were firmly resolved that we were gonna vaccinate"; “I was always very clear about vaccinations*.*”* However, one mother illustrated how easy it is for doubt or hesitancy to potentially take root:

"I do look at some of the arguments that people have for not vaccinating and I think there's a lot of people who think the same thing, I kind of wonder why. Like is there an actual reason? …Is it something there that we're really not seeing?"

THEME 4: INFORMATION BALANCE (GOLDILOCKS INFORMATION). The participants spoke a great deal about the importance of receiving or being able to find what they perceived as the “right” information–the right amount, from the right sources, at the right time, and about the right topics. Everyone had slightly different ideas about this ideal balance of information, however. As one parent explained, *“Some people feel like that information lacking is like someone’s hiding something or they’re not sharing all of the information*. *Whereas*, *you know*, *I like*, *here’s a simple summary but here’s some more information if you*, *you know*, *you’re one of these people that likes to read that stuff*.*”*

Some participants noted that it was particularly hard to find information on certain topics, such as getting vaccines outside the standard schedule. When they were able to get information, it was important that it came at the right time: *“If I’d known in advance that was a risk*, *you know*, *I might’ve said like well*, *you know*, *like can we delay it*?*”* And sometimes they didn’t want to hear about vaccination at all: *“I haven’t got time to read this or the headspace”*.

THEME 5: TRUST AND THE MESSENGER. Parents frequently discussed the critical role that trust plays in their experiences of communication about vaccination. Healthcare provider competence and knowledge were important factors in building trust, as was seeing the same provider over time: *“I feel like I do trust [the maternal child health nurse] because I feel like I know her*.*”* Participants saw information delivered by a trusted professional as more reliable, and the communication experience was more positive overall. But as one parent highlighted, if trust is broken for any reason, it is often irreparable: *"From that point once that trust is broken I think you need to find a new health professional*.*"*

Some parents described having different reactions to communication that came from peers, or from outside the healthcare encounter. One mother described the emotional impact of an online whooping cough campaign: *“That was so powerful and so community driven*, *it was a family that was taking action ‘cause what had happened to them*. *That was really different and very non-traditional*, *there was no government plan behind it or anything like that*.*”* Other participants noted that trust might be easier to establish during casual playground *“swing-pushing conversations”* between parents: *"Mums trust other mums a bit more than they trust health professionals*.*"* However, as this participant went on to say, *"I’m still a little bit sceptical about other people’s experiences*. *I’m interested in hearing about other people’s experiences but then I make up my own mind*.*"*

THEME 6: THE TAKEAWAY. Communication encounters themselves may be brief, but the focus group participants indicated that they continue to think about and reflect on these encounters after they have ended. The takeaway, or how the parent feels after a communication experience, can be just as important as the experience itself, and can shape future decisions. Parents described positive encounters as those where they were left feeling like they had been respected, supported, and were in control: *“I want to feel that I have made the decision*.*”*

In contrast, after a poor communication experience with a provider who was judgemental and did not adequately address her concerns, a participant said *"I felt a bit dismissed ‘cause I felt like*, *you know*, *she was sort of saying you shouldn’t have any emotions about it at all*.*"* A negative takeaway from a communication experience can have long-lasting ramifications, because it can shut down avenues for future conversation. Several parents mentioned changing healthcare providers following a negative experience, and after one particularly judgmental discussion with another mother, a participant said *“I probably would choose not to bring up my vaccination decisions with her again*.*”*

#### Professional themes

We identified five themes from the focus groups with professionals. The professionals discussed their experiences with vaccination communication in terms of their involvement in communication design, delivery or evaluation. They were generally familiar with the concept of outcomes, so their discussions also included explicit brainstorming of potential outcomes.

THEME 1: DESIGNING AND SELECTING INTERVENTIONS. While the goal of most of the professionals was to implement effective, evidence-based communication interventions, they acknowledged that there is a range of external factors that influence how interventions are designed and delivered. These factors include the information needs of their target audience, and resources: *“We were also driven by a timeline and a budget”*.

However, one international participant described a less obvious potential ulterior motive shaping a national communication strategy: *“Sometimes governments don’t want to take the responsibility and they are creating you know this community involvement* …*sometimes we use this communication strategy to send some hidden agenda*.*”*

In addition to choosing which communication interventions to implement, many of the professionals were involved in training and tailoring interventions to target key populations: *“The strategy that we came up with…wasn’t*, *you know*, *the government saying you need to immunise your child*, *it was an appropriately trained Aboriginal health worker*.*”*

THEME 2: PERCEPTIONS ABOUT WHAT PARENTS EXPERIENCE, WANT AND NEED. In designing and implementing communication interventions, the professional participants often tried to predict or speculate on what parents may want or need from a communication encounter. Healthcare providers and researchers, who are likely to interact directly with parents more frequently than policymakers, had the most to say on this theme.

Parents were seen as seeking help with the decision-making process from health providers: *"They’re struggling with the decision*, *they’re three quarters of the way there usually*, *they just need someone else to say it’s OK I’ll help you make this decision and do it in a safe environment"*, though cultural beliefs and social circles also shaped their decisions.

Many professionals agreed that parents want to feel respected, reassured, supported and in control, and they hoped that the vaccination communication they implemented would foster these feelings: *"When we were reaching out to them [we hoped] that they felt that they were important* …*and they're cared about"; "[I hope that] they get the vaccine*, *they feel happy about it*, *they’ve had all their questions answered*, *they’re accepting it*, *they’re satisfied with the encounter*.*"*

THEME 3: PERCEPTIONS ABOUT WHAT MAKES A GOOD COMMUNICATOR OR COMMUNICATION ENCOUNTER. In addition to speculating about what parents want from a communication encounter, the professionals reflected on the qualities and actions they felt make a good communicator.

According to the healthcare providers, projecting confidence and competence are critically important skills to reassure parents: *"I like to let them know that I'm absolutely in charge of what's going on*, *that I'm going to tell them where to sit or how to hold the baby and that I'm in control of the situation*.*"* Many of the healthcare providers described other nonverbal communication-related skills and behaviours that they felt improved the encounter, such as *"active listening"*; *"[being] able to pick up body language";* and *"[making] the next appointment with myself so that there’s some sort of continuity*.*"*

Most of the professionals agreed that the ideal communication encounter builds and maintains trust. Maintaining an ongoing positive relationship was seen as particularly important with parents who refuse vaccines, so that *"if they change their mind they feel they can go back to that clinician*.*”*

THEME 4: CHALLENGES IN A COMMUNICATION ENCOUNTER. While the professional focus group participants were aware of the features of a good communication encounter, putting these factors into practice could be challenging. One major obstacle mentioned by all the providers was time pressure. Building a trusting relationship requires patience, so that parents don't feel rushed. One provider said that she tried as much as possible *"to communicate that I have all the time in the world*,*"* but as another said, *"it's hard sometimes 'cause we don't have the time*.*"*

Echoing some of the sentiments raised by parents, the providers discussed the importance and difficulty of delivering the right amount of information without overloading parents: *"Sometimes when we talk too much or give too [much] information*, *that's overwhelming for parents and they get lost*.*"* They also noted the difficulties that have arisen as parents increasingly seek information from alternative sources, such as the internet: *"So many parents are using Doctor Google and coming up with really horrible things*.*"*

THEME 5: "IT'S VERY DIFFICULT TO MEASURE": EVALUATION CHALLENGES. Because the goal of these focus groups was to identify outcomes to measure the effects of vaccination communication, the professionals spent a significant portion of each session discussing their experiences with intervention evaluation and the challenges this can present.

The researchers discussed the balance between choosing outcomes important to parents, and those important to public health: *"But in the bottom line*, *if everyone felt happy about their decision but the uptake was 70 percent*, *I’d prefer to have an uptake of 95 percent with not everyone happy with the decision*.*"* Vaccination rates are obviously important, but there is debate about the best specific vaccination-related outcome to select: *"If we're choosing between completion at six weeks versus one year*, *are we saying that one particular outcome is more important than the other*?*"* Political or financial factors can also influence the selection of outcomes: *"There [could be] conflicts of interest in what they are doing*. *Somebody's paying for something and is interested in some outcome*.*"*

Sometimes, selecting an outcome in principle is more straightforward than actually defining and operationalising it for the purposes of an evaluation. This may be because there is no available data, no appropriate measurement tool, insufficient funding for evaluation, or confusing terminology: *"Community engagement*, *community involvement*, *community participation*, *what do they all mean*?*"*

### Stage 2: Interpretive analysis results–translating themes into outcomes

In our second analysis stage, we translated the thematic ‘experiential’ data from focus groups into language compatible with the language used in the outcomes research literature, so they could be considered for COS prioritisation (see S3 Appendix for full list of themes and codes). For example, parent theme 1 (Concerns and fears) included a number of specific codes, such as “Concerns about reactions or side effects” and “Concerns about stress or pain of vaccine delivery”. Through comparison with the Map, we identified correspondence between the codes for this theme and consumer-related outcomes including anxiety, attitudes or beliefs, and risk perception.

Through this process, we identified 47 outcomes raised by parents. Table 2 presents the parent themes (left column) and corresponding translated outcomes (right column). Not every topic discussed by the participants could be translated into an outcome, while some generated multiple outcomes.

**Table 2:** Parent themes and translated outcomes.

PARENT THEMES	OUTCOMES TRANSLATED FROM THEMES AND CODES
1. Concerns or fears	- Attitudes or concerns about vaccination or vaccines - Attitudes or concerns about reactions or side effects - Perceived risk of side effects or pain - Attitudes or concerns about pain of vaccine delivery - Attitudes or concerns about vaccine safety - Attitudes or concerns about diseases - Anxiety or stress related to vaccination - Confidence in efficacy or safety of vaccinations
2. Making a choice	- Subjective norm (perceived social pressure to engage in behaviour) - Perceived risk and severity of diseases - Amount and appropriateness of decision support received
3. Certainty and confidence	- Confidence in planned decision - Confidence in ability to stay on schedule (behavioural control/self-efficacy) - Clarity of values - Timeliness/ on-time vaccination
4. Information balance	- Acceptability of intervention content and/or design - Satisfaction with intervention topic and content - Knowledge about where and how to find relevant information - Confidence in ability to find or understand information - Confidence in ability to judge information quality - Knowledge about judging information quality - Perceived quality of intervention content - Perceived accuracy of intervention content - Clarity of intervention - Accessibility or readability of intervention - Satisfaction with quantity of information - Anxiety or stress related to intervention - Satisfaction with timing of intervention delivery - Time taken to deliver/receive intervention - Satisfaction with intervention format - Cultural appropriateness of intervention - Level of information seeking or avoidance
5. Trust and the messenger	- Satisfaction with intervention delivery - Perceived competence of communicator/ provider - Provider knowledge about vaccination, schedule, diseases - Perceived knowledge of communicator/ provider - Perceived support given by communicator/ provider - Confidence in communicator/ provider's skills and knowledge - Trust in communicator/ provider - Continuity of provider
6. The takeaway	- Perceived control over decision-making process - Degree of involvement in the decision-making process - Perceived influence of intervention on decision taken - Satisfaction with decision support - Confidence in decision-making ability - Patient-centredness of encounter - Satisfaction with the decision process

In total, we identified 73 outcomes raised by the professionals (Table 3). As for parents, we translated the professional themes and codes into outcomes. For instance, professional theme 3 (Perceptions about what makes a good communicator or communication encounter) included a contributing code, ‘“Every opportunity’s a good opportunity” to discuss or deliver vaccines'. We translated this code into the outcome of missed or captured opportunities. In addition to describing their experiences with vaccination communication interventions and evaluations, some of the professional participants explicitly brainstormed or discussed outcomes in their focus groups. This meant that some outcomes were recorded directly from the professional transcripts, without requiring translation to link a thematic concept with an outcome. These outcomes appear at the end of the table.

**Table 3:** Professional themes and translated outcomes.

PROFESSIONAL THEMES	OUTCOMES TRANSLATED FROM THEMES AND CODES	
1. Designing and selecting interventions	- Political acceptability of intervention - Cultural appropriateness of intervention - Accessibility or readability of intervention - Acceptability of intervention - Clarity of intervention	- Degree of outreach or engagement in intervention design and delivery
2. Perceptions about what parents experience, want and need	- Attitudes or concerns about reactions or side effects - Perceived risk of side effects or pain - Anxiety or stress related to vaccination - Anxiety or stress related to intervention - Subjective norm (perceived social pressure to engage in behaviour) - Perceived support given by communicator/ provider - Amount and appropriateness of decision support received - Satisfaction with the decision-making process	- Confidence in decision made - Perceived control over the decision-making process - Confidence in ability to stay on schedule (behavioural control/self-efficacy) - Parent behavioural control/self-efficacy to make decisions - Satisfaction with intervention topic or content - Satisfaction with intervention delivery
3. Perceptions about what makes a good communicator or communication encounter	- Provider communication skills self-efficacy - Provider confidence in own communication skills - Provider confidence in ability to participate in shared decision-making - Provider knowledge about vaccination, schedule, diseases - Provider knowledge about how to find additional information	- Provider knowledge about communication issues - Trust in communicator/ provider - Confidence in communicator/ provider's skills and knowledge - Missed/captured opportunities to discuss, plan or deliver vaccination - Continuity of provider
4. Challenges in a communication encounter	- Satisfaction with quantity of information - Time taken to deliver/receive intervention	- Provider satisfaction with timing of intervention
5. "It's very difficult to measure": evaluation challenges	- Impact or reach of intervention	- Cost or resource use of intervention
Outcomes recorded directly from transcripts	- Attitudes or concerns about vaccination or vaccines - Intention to vaccinate - Vaccine acceptance - Vaccine hesitancy - Community awareness about available vaccination services and organisations - Source of vaccination information - Whether plans are implemented as intended - Functionality of community vaccination organisations - Number of meetings of community organisations - Number visits to health facilities by ward committees - Number of house visits by community health workers - Cost-effectiveness of interventions - Clarity of values - Decisional conflict - Regret with decision made - Perceived risks of disease Shared decision-making - Parents feeling proactive or like they've taken a positive step - Vaccination consent card return rate - Timeliness	- Time spent undervaccinated - Unintended impacts of interventions Satisfaction with the decision process - Deaths from vaccine-preventable diseases - Knowledge and awareness of vaccination services - Vaccination objection or exemption rate - Knowledge about judging information quality - Knowledge about where and how to find relevant information - Being up-to-date with knowledge - Parent knowledge of vaccine schedule - Provider confidence in their own knowledge - Parents feeling supported by their community - Parents feeling important or valued in a communication encounter - Use and reach of information or intervention - Anticipated regret - Degree of involvement in the decision-making process - Attendance at appointments or health facilities - Number of vaccines delivered - Uptake or coverage

We combined the parent and professional outcomes, removed duplicates, and compared this list of focus group outcomes with the trial outcomes mapped from our earlier literature review (S4 Appendix). We found that were 57 distinct outcomes measured in trials, while 91 total outcomes were raised in the focus groups. Of the focus group outcomes, 61 (67%) had not been measured in trials, with most of the new outcomes arising in the areas of attitudes or decision-making.

## Discussion

### Summary of findings

In this paper, we have presented the methods and findings of a focus group study involving parents of young children and health professionals involved in vaccination communication. These focus groups formed the stakeholder consultation step undertaken prior to outcome prioritisation in the process of COS development [20, 21].

Thematic analysis yielded six themes related to the experiences of parents and five themes related to professionals’ experiences. Parental experiences revolved around making the decision about vaccination. They discussed their anxieties and concerns, and feelings of certainty or confidence about their options and planned course of action. Their varied preferences highlighted the often personal balance of factors at play, such as the quantity and timing of information, a finding echoed by related qualitative studies about communication preferences [33]. They largely agreed on the qualities that made a good communicator and how a good communication experience should make them feel. The experiences and perspectives of the professionals were less personal than those discussed by the parents. The professional themes loosely centred around three aspects of the intervention process: planning, implementation, and evaluation.

We then interpretively analysed the focus group data to translate them into 47 potentially measurable outcomes raised by parents and 73 raised by professionals (91 total outcomes, with duplicates removed).

### Comparing focus group outcomes and research literature outcomes

Two thirds of the outcomes raised in the focus groups had not been measured in trials [18]. This demonstrates the need for and value of looking to stakeholders to identify outcomes based on their experiences, in addition to identifying outcomes from research literature. Most of these new focus group outcomes were related to attitudes or beliefs and knowledge. For example, the focus group participants raised several outcomes associated with confidence: confidence in their ability to find or judge the quality of information (both parents and providers), confidence in their own knowledge and provider confidence in their communication skills. The new knowledge outcomes they raised included knowledge about finding relevant information and judging information quality. Several community participation outcomes related to measuring the functionality of a vaccination-related community health organisation were also uniquely contributed by focus group participants.

Further research is required to develop and validate measurement tools or scales for some of the focus group-generated outcomes.

### Comparing parent and professional outcomes to explore how they think about ‘successful’ communication

There was some overlap between the outcomes raised by parents and professionals, particularly in the areas of attitudes or beliefs and decision-making. They had very little overlap in their discussion of community participation outcomes or outcomes related to vaccination status and behaviours.

Based on the outcomes they raised, successful vaccination communication, for parents, impacts how they feel and how they make decisions–but not necessarily what they do. This is supported by the parent themes, which focused on aspects of the experience such as anxiety, choice, trust and feelings during and after communication encounters. The vast majority of the outcomes parents raised (35 of 47 total outcomes) were related to attitudes or beliefs and decision-making. However, only two outcomes from the parent focus groups were associated with actual vaccination behaviours or practices. Notably, parents did not mention their own knowledge levels about vaccination issues–the only types of knowledge they discussed were their provider's knowledge and their knowledge about finding and judging information on their own.

The professionals discussed more outcomes than parents (though there were also more professional participants). Outcomes raised by the professionals included a broader range of health systems-related outcomes, such as cost-effectiveness of the intervention and appointment attendance. Perhaps predictably, the actual vaccination-related behaviours of parents were important to professionals, as were public health or health systems concepts such as cost and health status. However, the analysis suggested that professionals were conscious that parent attitudes and decision-making were key areas to influence in order to achieve these external goals: like the parents, most of the outcomes raised by the professionals related to these two categories. The two themes related to professional perceptions of parents' experiences and the communication encounter also support this interpretation. Professionals also raised many more outcomes associated with knowledge than the parents. This could indicate that some professionals still think and operate according to a deficit model of knowledge, i.e. if parents are educated and informed about vaccination they will change their behaviour [34, 35]. However, several of the knowledge outcomes raised by professionals were focused on provider knowledge levels, which suggests a different and perhaps more nuanced awareness of the importance of provider-parent communication in the parent decision-making process [36, 37].

### Strengths and limitations of the study

This study focused specifically on childhood vaccination, because the delivery schedule is most intense and vaccination communication encounters most frequent during the first few years of a child’s life. However, many of the outcomes raised by participants in these focus groups are likely to be applicable across settings, populations and even communication topics. For instance, many participants discussed important process outcomes associated with communication delivery and satisfaction, or knowledge outcomes associated with finding and judging the quality of health information. Although this has not been formally assessed, the outcomes from these focus groups and in the COS itself are therefore also likely to be highly relevant for studies of communication targeting adolescent and adult vaccination.

#### Participants

The range of professionals consulted (researchers, policymakers and providers) was a strength of this research, because providers are often the primary or sole professional group consulted in COS development studies. However, recruitment difficulties and resource availability limited the total number of participants and sessions held. We faced logistical challenges finding times and locations that suited the participants, as both health professionals and parents of young children are particularly busy groups to coordinate. As a result, some focus groups had fewer participants than we had intended to include, though evidence about the impact of small group size is mixed [22]. We were not able to recruit any physicians for the healthcare provider focus group, so only nurses were included. A more diverse group may have raised some additional outcomes, but the fundamental features of the parent-provider communication encounter are likely to be similar regardless of provider. Furthermore, there were some doctors who provided input through other focus groups, albeit as representatives from their other professional stakeholder role (e.g. researcher or policymaker).

Some of the sessions involved 'natural' groups, where the participants knew one another. Natural groups have some potential advantages, such as participants feeling more comfortable and possibly opening up or interacting more than when amongst strangers, but they may also have disadvantages in that participants may adhere to established social norms and may be less likely to express dissenting opinions [38, 39]. From an observational perspective, these sessions did not appear different from the other focus groups in terms of participant interactions or opinions expressed, but there may have been unseen influences.

Most of the participants were based in Australia, with the exception of the international policymakers, but it is not possible to determine the implications of the stakeholder locations. One professional focus group was held over teleconference, which may have reduced the spontaneity of the conversation, though the interaction level in that session was roughly similar to that in the other sessions.

While the aim of the parent recruitment process was to include participants from a variety of socio-economic and cultural backgrounds, the parents who ultimately participated in the study were all women and were demographically relatively homogenous. The overrepresentation of women in these parent focus groups is common in research about childhood vaccination, and reflects the fact that mothers are more commonly identified as the primary healthcare decision-maker for their children [40]. The impact of diversity of socioeconomic status, linguistic background or education level on the outcomes raised is not known and would be a valuable topic for further research, as these populations are often the target of vaccine promotion efforts.

#### Analysis

This is the first known effort to explore parent and professional perspectives on vaccination communication specifically in terms of potentially measurable outcomes. A major challenge we faced, which has been shared by other COS researchers [20], was determining how to discuss the concept of vaccination communication outcomes with participants for whom this is not a familiar or easily understood topic. We addressed this issue primarily through the two-stage analysis method (i.e. thematically analysing the transcripts and then translating the themes and codes into outcomes), rather than by trying to explain the concept of outcomes in the discussions themselves. The advantage of this approach was that participants were able to speak freely about their experiences, a topic with which they were comfortable and familiar. By translating the outcomes into the language used by researchers, however, we sacrificed the specific language of the participants, which can be valuable to carry through to the COS prioritisation survey to help the survey participants to recognise and understand the outcomes they are rating [20]. Nevertheless, this translation was necessary, because as leading methodological experts Pope and Mays write, "until something is classified it cannot be measured" [41]. Translation into outcome evaluation language enabled us to integrate the focus group outcomes with those derived from trials and health communication literature to develop a comprehensive taxonomy of outcomes [42] that were prioritised in a Delphi survey [43]. Furthermore, establishing a consistent way to describe and define outcomes in this area, which has been characterised by disparate and inconsistent language, was an important goal of this project as a whole.

Another potential limitation of this study was the fact that the outcomes raised by parents and professionals were relevant to their experiences, but not necessarily those that were considered 'important to' or 'valued by' the stakeholders. Therefore, using these outcomes to make inferences about stakeholders’ views on ‘successful’ communication is an interpretation that may, in part, reflect researcher perspectives.

### Reflexivity

Throughout the study process, the authors were aware of our own positions and reflected on how these could influence the study design, conduct and analysis. All three authors work with the Cochrane Consumers and Communication group, so we support the informed involvement of patients in healthcare, and we are also advocates for childhood vaccination. It is possible that our perspectives as researchers in favour of vaccination may have influenced the manner in which we collected and interpreted the data or the implications for future research and practice which we have drawn.

## Conclusion

This was the first known study to explore how parents and professionals experienced vaccination communication, identify those aspects of the experience that were important to them, and translate these into outcomes. We found that most outcomes raised in the focus groups were not measured in existing vaccination communication trials. There may be several reasons for this, including lack of known or validated measurement methods and limitations on the number of outcomes a given trial can assess. However, lack of awareness on the part of researchers is another key contributor to the relatively narrow range of outcomes generally selected for trial evaluations. This study provides valuable insight into what different stakeholders want and value from a vaccine communication intervention: outcomes related to attitudes or beliefs and decision-making were discussed most frequently and are a shared concern of parents and professionals. The outcomes identified in this study can be integrated with additional outcomes from vaccination and health communication research for organisation into a taxonomy [42], prioritisation into a Core Outcome Set [43], and assessment in trials.

## Supporting information

S1 Appendix Focus group discussion guides. (DOCX)

S2 Appendix Focus group visual aids. (PDF)

S3 Appendix Focus group themes and codes. (DOCX)

S4 Appendix Comparison of outcomes measured in trials and raised by focus group participants. (DOCX)
