# Supplementary material for: Qualitative focus groups with stakeholders identify new potential outcomes related to vaccination communication
Source: PLoS One. 2018 Aug 1;13(8):e0201145. doi: 10.1371/journal.pone.0201145 (PMC6070264; doi:10.1371/journal.pone.0201145)
Supplement: S1 Appendix — (DOCX) [file pone.0201145.s001.docx]

# S1 Appendix. Focus group discussion guides

## Professional focus group discussion guide

### Registration

- *Distribute consent form*
- In this focus group, you will be discussing your experiences and perspectives as a [professional role] on vaccination communication issues. Your participation in this study is completely voluntary, and you can stop participating at any time by leaving the session, though you will not be able to withdraw any information you have already provided.
- The session will be audio recorded and transcribed but your comments will be kept confidential – all the participants will use only each other’s first names, and you will be assigned an alias in any transcripts or analyses written after the session.
- You will be given a $30 gift voucher to cover your time and travel expenses at the conclusion of the session.
- Do you have any questions? Do you consent to participate in this study? Please sign this consent form.
- *Collect signed consent forms*
- *Offer optional copies of Participant information sheet or the focus group background*
- *Distribute name tags*

### Welcome and project overview

- Introduction
- In this focus group, you will be discussing your experiences and perspectives as a professional (even if you have children). Your participation in this study is completely voluntary, and you can stop participating at any time by leaving the session, though you will not be able to withdraw any information you have already provided.
- The session will be audio recorded and transcribed but your comments will be kept confidential – all the participants will use only each others’ first names, and you will be assigned an alias in any transcripts or analyses written after the session. Please talk to and respond to one another
- PhD and study
- I’m working on a project called ‘Communicate to Vaccinate’ which is an international research project looking at different ways to improve communication about childhood vaccination around the world. This study in particular will be a part of my PhD, which aims to improve the way communication for vaccination is evaluated and determine what “effective” communication should look like and achieve.
- We are conducting a series of focus groups with different stakeholders, including parents, health providers, researchers and policy makers. The aim of these focus groups is to discuss potential outcomes that are relevant to each group.

### Introductions

- Tell us your name and briefly describe your role and how you are involved in vaccination and vaccination communication OR describe any experience you’ve had with vaccination communication evaluation

### Vaccination communication interventions

- We are here today to talk about communication about vaccination.
- Like a medication or a therapy, communication is something that can impact people’s health, and it can be studied and measured. It involves much more than just a pamphlet or a doctor speaking to a patient. We include a lot of different things in our definition of “communication.”
- In the COMMVAC project, we created a taxonomy of communication interventions related to childhood vaccination, which we organised by their purpose. Our focus is on communication that directly impacts parents or communities, which includes communication directed to these people or groups but also some communication targeted to health professionals. Here are some examples of the PURPOSES of communication (show visual aids)

#### Discussion about interventions

- Think of a particular instance where you were engaged in communication about vaccination. Describe that experience.
  - What happened?
  - Did it go the way you hoped it would?
  - Do you think it “worked” based on what you think it was trying to achieve?
  - Has anyone had a different experience with the same kind of communication?
- What are some other kinds of communication related to vaccination that you have used or know of?
  - For example, reminders, facebook forum, video, decision aids
  - Do you tailor your communication for different purposes (eg new vaccine introduction) or populations (eg hesitant parents, new migrants)
  - Do you tend to rely on one route or medium for communication? Or use multiple
- Who is involved in designing the communications?
  - Do you have any choices in which communication strategies or tools you use?

### Vaccination communication outcomes

- We can see that there are lots of different types of communication interventions. Communication can also have different effects or outcomes. Outcomes might be a particular behaviour like someone going to get a vaccine for their child, or a feeling of stress or anxiety about the decision or satisfaction with how the information was delivered.
- Sometimes different people are interested in different outcomes - for instance, the head of the department funding an information campaign may only want to know how many more children have been vaccinated since starting that campaign. But a nurse might want to know whether the parent understands the message or feels confident in their decision, and a parent might want to feel like they have been listened to and respected in their interaction, and supported to make their decision.
- These things are all measurable outcomes, but a lot of the time only some are being measured.

#### Discussion about outcomes

- Have you ever been involved in an evaluation of a vaccination program or of a communication strategy?
  - What types of outcomes did you measure?
- Other than improving vaccination coverage, are there outcomes you would like to achieve with your communication?
  - How do you hope it will make parents/communities or providers feel or behave?
- Do you ever look at research evidence to help decide what strategy to use or to find out what options might be possible?
  - If you were looking at a study of a communication strategy to see if it might work in your setting or population, what outcomes would you focus on or be looking for?
  - What outcomes are most important to you, in your professional role?
- What are some barriers to measuring outcomes?
- If there were no constraints, what is a communication intervention you would like to be able to test or implement, and what kind of outcomes would you like to measure to evaluate it?
- What other outcomes do you think COULD or SHOULD be measured, either routinely or in certain situations or populations, based on the range of interventions we have discussed?
- You’ve talked about your experiences with communication today, and we have been listening in particular for what kinds of outcomes you’ve indicated might be important. So from what you’ve said, a few things we might want to measure to find out if communication is effective are: (examples from notes)
- Is there anything else you think we should consider measuring to evaluate communication?

### Wrap up

- Thank you!
- We will analyse your comments today to identify some key themes in regards to what makes an effective communication intervention. We will be combining this information with the input from health providers, parents and researchers as well. This will help the people who are designing, delivering and evaluating communication interventions.
- We would love for you to be involved in an online survey that will take place after we finish all the focus groups, and will give you a chance to take part when it’s up and running
- *Questionnaire*
- *Gift certificate*
- *Receipt of payment*

## Parent focus group discussion guide

### Registration

- *Distribute consent form*
- In this focus group, you will be discussing your personal experiences and perspectives on vaccination communication issues. Your participation in this study is completely voluntary, and you can stop participating at any time by leaving the session, though you will not be able to withdraw any information you have already provided.
- The session will be audio recorded and transcribed but your comments will be kept confidential – all the participants will use only each other’s first names, and you will be assigned an alias in any transcripts or analyses written after the session.
- You will be given a $30 gift voucher to cover your time and travel expenses at the conclusion of the session.
- Do you have any questions? Do you consent to participate in this study? Please sign this consent form.
- *Collect signed consent forms*
- *Offer optional copies of Participant information sheet or the focus group background*
- *Distribute name tags*

### Individual exercise

- *Post-it notes available at each seat*
- While we wait for everyone to arrive, you’ll see some post-it notes at your seat. Please think and write about an experience you had with communication about vaccination to share.
  - How did you feel before the experience?
  - What happened?
  - How did you feel after, and do you think it achieved what it was aiming to achieve?

### Welcome and project overview

- Introduction
- In this focus group, you will be discussing your experiences and perspectives as a professional (even if you have children). Your participation in this study is completely voluntary, and you can stop participating at any time by leaving the session, though you will not be able to withdraw any information you have already provided.
- The session will be audio recorded and transcribed but your comments will be kept confidential – all the participants will use only each others’ first names, and you will be assigned an alias in any transcripts or analyses written after the session. Please talk to and respond to one another
- PhD and study
- I’m working on a project called ‘Communicate to Vaccinate’ which is an international research project looking at different ways to improve communication about childhood vaccination around the world. This study in particular will be a part of my PhD, which aims to improve the way communication for vaccination is evaluated and determine what “effective” communication should look like and achieve.
- What we want to find out from these focus groups is what kinds of things are important to you, as parents, when you are involved in communication about vaccination. In other words, what do you think makes communication "effective" or "not effective"?

### Introductions

- Going around the table, tell us your name, how many children you have, and how old they are

### Sharing personal experiences

- Share the personal experiences from post its
- What happened?
- How did it make you feel?
- Do you think it “worked” based on what you think it was trying to achieve?
- Has anyone had a different experience with the same kind of communication?

### Vaccination communication interventions

- Like a medication or a therapy, communication is something that can impact people’s health, and it can be studied and measured. It involves much more than just a pamphlet or a doctor speaking to a patient. We include a lot of different things in our definition of “communication.” We find it helpful to think about them based on the purpose of the communication. Here are some examples of the PURPOSES of communication (show taxonomy category visual aid if using).

#### Discussion about other types of communication

- Has anyone had an experience with any of these other types of communication that you’d like to discuss?
  - For example, reminders, facebook forum, video, decision aid
- (OPTIONAL DISCUSSION PROMPT): Were there ever aspects of the vaccination process that made you anxious or uncertain?
  - Do you think you looked for different things in the communication you experienced when you were anxious? Do you think your feelings about what is effective communication were different?

### Vaccination communication outcomes

- You’ve talked about your experiences with communication today, and we have been listening in particular for what kinds of outcomes you’ve indicated might be important. So from what you’ve said, a few things we might want to measure to find out if communication is effective are: (examples)

#### Discussion about outcomes

- Is there anything else you think we should consider measuring to evaluate communication?
- In an ideal communication situation, how would you like it to make you feel? What do you think its goals should be?

### Wrap up

- Is there anything else you would like to add or clarify before we wrap up?
- Thank you!
- We will analyse your comments today to identify some key themes in regards to what makes an effective communication intervention. In the next stage of the research, we will find out which outcomes are important to health providers, policy makers and researchers as well. This will help the people who are designing, delivering and evaluating communication interventions.
- We would love for you to be involved in an online survey that will take place after we finish all the focus groups, and will give you a chance to take part when it’s up and running
- *Questionnaire*
- *Gift certificate*
- *Receipt of payment*
