# Supplementary material for: Qualitative focus groups with stakeholders identify new potential outcomes related to vaccination communication
Source: PLoS One. 2018 Aug 1;13(8):e0201145. doi: 10.1371/journal.pone.0201145 (PMC6070264; doi:10.1371/journal.pone.0201145)

S2 Appendix. Focus group visual aids

Communication interventions related to childhood vaccination

PURPOSE \ TARGET	Parents or soon-to-be parents	Communities, community members or volunteers	Health professionals
**Inform or Educate**	One on one interactions; Device, tool or object; Group interactions; Audio visual / performance; Mail or email; Printed material; Phone-based; Web-based	One on one interactions; Device, tool or object; Celebrity spokespeople; Group interactions; Audio visual / performance; Mass media campaign; Mail or email; Printed material; Community event; Phone-based; Web-based	Phone-based
**Remind or Recall**	One on one interactions; Phone-based; Mail or email; Device, tool or object		One on one interactions; Device, tool or object
**Teach Skills**	Training in how to communicate / educate others; Parenting skills programs	Training in how to communicate / educate others	Training in how to communicate / educate others
**Provide Support**	One on one interactions; Phone-based; Group interactions; Web-based		
**Facilitate Decision Making**	Decision aids		Decision aids
**Enable Communication**	Interpreters		
**Enhance Community Ownership**	Program delivery	Local opinion leaders; Program delivery; Partnership building; Community input; Community coalition	

Inform or Educate

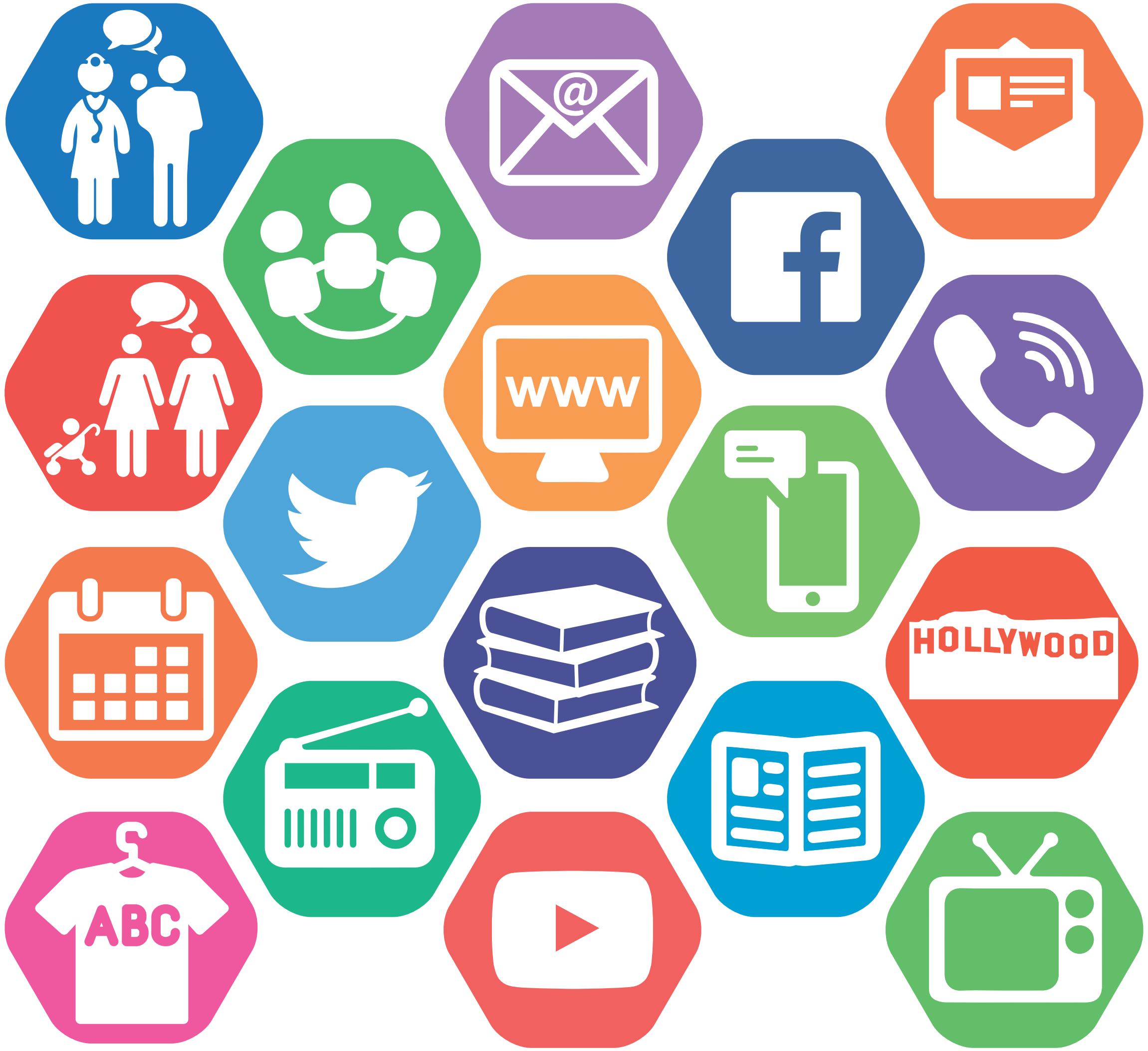

Remind or Recall

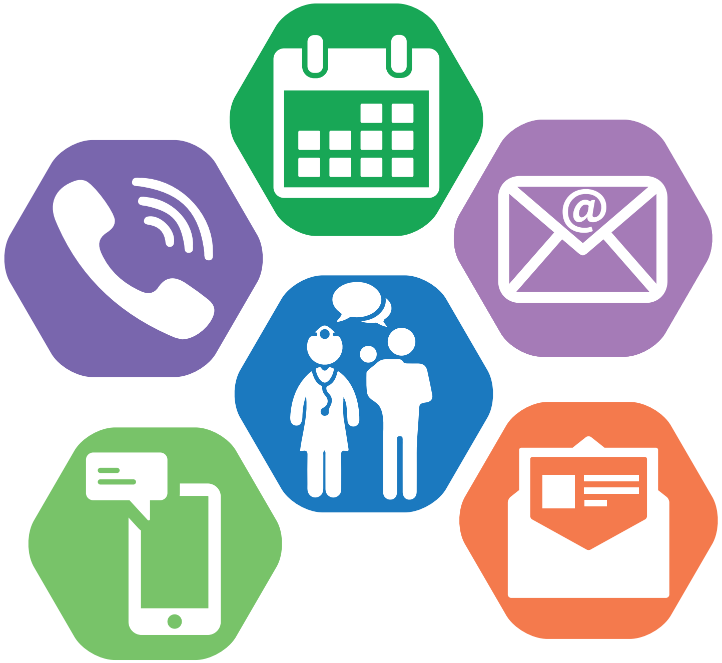

Teach Skills

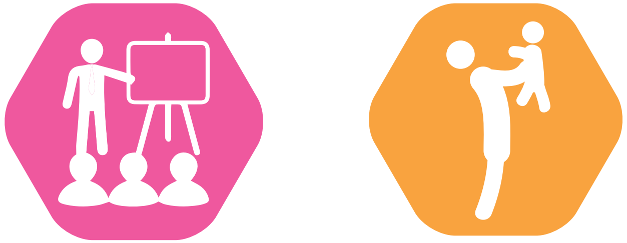

Provide Support

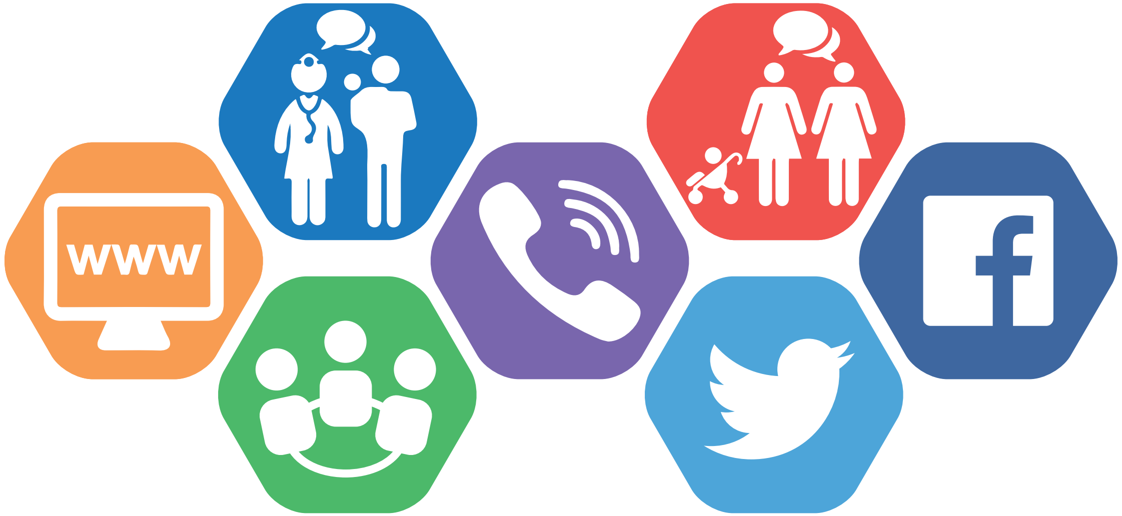

Facilitate Decision Making

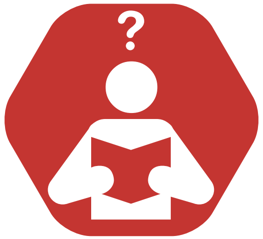

Enable Communication

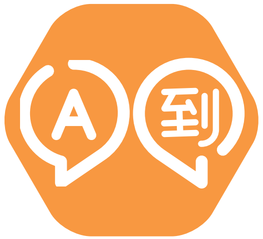

Enhance Community Ownership

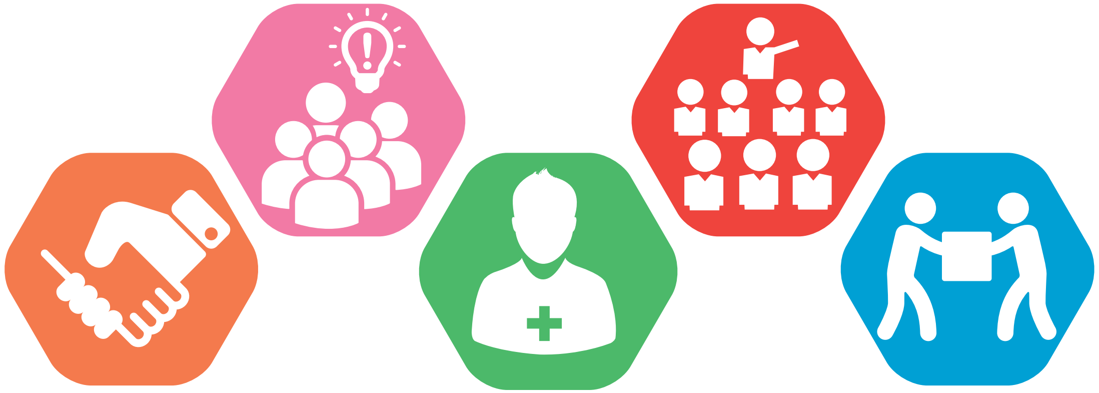

Supplement: S2 Appendix — (PDF) [file pone.0201145.s002.pdf]
