# Supplementary material for: Qualitative focus groups with stakeholders identify new potential outcomes related to vaccination communication
Source: PLoS One. 2018 Aug 1;13(8):e0201145. doi: 10.1371/journal.pone.0201145 (PMC6070264; doi:10.1371/journal.pone.0201145)
Supplement: S3 Appendix — (DOCX) [file pone.0201145.s003.docx]

# S3 Appendix. Focus group themes and codes

**Parent focus group themes and specific codes**

| 1. Concerns or fears    1. About reactions or side effects    2. About stress or pain of vaccine delivery    3. About vaccine safety    4. About vaccine-preventable disease    5. “It's different after your first child” |
| --- |
| 1. Making a choice    1. Your background shapes your perspective    2. Seeing the disease can influence your decision    3. Looking for guidance about what to do    4. People should be allowed to weigh the options and decide for themselves |
| 1. Certainty and confidence    1. Feeling uncertain about vaccination    2. Feeling confident about vaccination    3. Uncertainty or confidence in parenting responsibilities related to vaccination |
| 1. Information balance    1. Knowing how and where to find information for yourself    2. Gauging information quality    3. Clear, understandable and accurate information    4. The right amount of information at the right time    5. Balancing feeling rushed with taking too long    6. Personalised or targeted communication    7. Actively blocking or avoiding vaccination information or discussions |
| 1. Trust and the messenger    1. A competent person delivering the vaccine itself improves the communication experience    2. Continuity of the communicator can impact communication    3. Trust is essential but it's hard to build and easy to break    4. "Swing-pushing" conversations - communication between parents is different from communication with a professional |
| 1. The takeaway    1. You want to feel like you're in control and your decision is respected    2. Good communication makes you feel supported    3. Poor communication makes you feel confused, dismissed or judged |

**Professional focus group themes and specific codes**

| 1. Designing and selecting interventions    1. Intervention design is influenced by external factors    2. Tailoring and targeting communication to engage different audiences |
| --- |
| 1. Perceptions about what parents experience, want and need    1. Parents have concerns about reactions or side effects    2. Parents are influenced by their own previous experiences and by other people    3. Parents want help with the decision-making process    4. Parents want to feel respected, reassured, supported and in control |
| 1. Perceptions about what makes a good communicator or communication encounter    1. Good communicators are confident, competent and in charge    2. Good communicators are knowledgeable and know where to find more information    3. Good communicators don't just talk    4. Good communication builds trust    5. “Every opportunity’s a good opportunity” to discuss or deliver vaccines |
| 1. Challenges in a communication encounter    1. Competing with Dr. Google    2. Balancing the right amount of information    3. Having enough time for communication |
| 1. "It's very difficult to measure": evaluation challenges    1. Selecting which outcomes to measure    2. Defining outcomes and measurement methods    3. Data availability and access limitations    4. Identifying the specific impacts of communication    5. Potential uncertainty or imprecision of using indicator or proxy    6. Funding and resource constraints for evaluations |
