# Supplementary material for: Qualitative focus groups with stakeholders identify new potential outcomes related to vaccination communication
Source: PLoS One. 2018 Aug 1;13(8):e0201145. doi: 10.1371/journal.pone.0201145 (PMC6070264; doi:10.1371/journal.pone.0201145)
Supplement: S4 Appendix — (DOCX) [file pone.0201145.s004.docx]

# S4 Appendix. Comparison of outcomes measured in trials and raised by focus group participants

**S4 Table A: Outcomes arising in both trials and focus groups**

|  | **TRIAL OUTCOMES** | **FOCUS GROUP OUTCOMES** | | |
| --- | --- | --- | --- | --- |
| **Outcome categories** | **Outcomes from the TRIAL OUTCOMES MAP** | **Outcomes raised by BOTH parents and professionals** | **Outcomes raised by PARENTS ONLY** | **Outcomes raised by PROFESSIONALS ONLY** |
| **HEALTH OR WELL-BEING** | Anxiety |  |  |  |
|  | - As a result of the communication intervention | - Anxiety or stress related to intervention |  |  |
|  | - About vaccination or vaccine-preventable diseases | - Anxiety or stress related to vaccination |  |  |
| **ATTITUDES OR BELIEFS** | Attitudes or beliefs |  |  |  |
|  | - About vaccination or specific vaccine/s | - Attitudes or concerns about vaccination or vaccines |  |  |
|  |  |  | - Attitudes or concerns about vaccine safety |  |
|  | - About communication interventions |  |  |  |
|  | Intention to vaccinate |  |  | - Intention to vaccinate |
|  | - Intended choice |  |  |  |
|  | - Likelihood of immunising on time |  |  |  |
|  | Evaluation of communication intervention |  |  |  |
|  | - Perceived effectiveness of the intervention |  |  |  |
|  | - Acceptance/acceptability of intervention | - Acceptability of intervention content and/or design |  |  |
|  | - Preference for intervention format or timing |  | - Satisfaction with timing of intervention delivery | - Provider satisfaction with timing of intervention |
|  | - Patient centredness of care |  | - Patient-centredness of encounter |  |
|  | - Use and reach of information or intervention |  |  | - Use and reach of information or intervention |
|  | - Response to information |  |  |  |
|  | Reason for vaccination or undervaccination |  |  |  |
| **DECISION-MAKING** | Decision-making |  |  |  |
|  | - Decision anxiety |  |  |  |
|  | - Decisional conflict |  |  | - Decisional conflict |
|  | - Decision satisfaction (with the choice, process, or preparation for decision-making) | - Satisfaction with the decision-making process |  |  |
|  | - Perceived behavioural control |  |  | - Parent behavioural control/self-efficacy to make decisions |
|  | - Perceived subjective norm | - Subjective norm (perceived social pressure to engage in behaviour) |  |  |
|  | - Risk perception | - Perceived risk and severity of diseases |  |  |
|  |  | - Perceived risk of side effects or pain |  |  |
|  | - Perceived outcome efficacy |  |  |  |
| **KNOWLEDGE OR UNDER-STANDING** | Knowledge or understanding |  |  |  |
|  | - About particular disease/s and related vaccines | - Provider knowledge about vaccination, schedule, diseases |  |  |
|  | - About risks, contraindications or side effects of vaccination or specific vaccines |  |  |  |
|  | - About vaccination schedule |  |  |  |
|  |  |  |  | - Parent knowledge of vaccine schedule |
|  | - About local vaccine services |  |  | - Knowledge and awareness of vaccination services |
|  | - About child health issues |  |  |  |
|  | Information retention over time |  |  |  |
|  |  |  |  |  |
|  | Intervention readability and comprehension | - Clarity of intervention |  |  |
|  |  | - Accessibility or readability of intervention |  |  |
| **VACCINATION UPTAKE AND BEHAVIOURS** | Receipt of vaccine / vaccination status |  |  | - Uptake or coverage |
|  | - Receipt of one or more vaccines |  |  |  |
|  | - Number of vaccines per child |  |  |  |
|  | - Number / proportion of children fully, partially or unvaccinated |  |  |  |
|  | - Vaccination dropout rate |  |  |  |
|  | - Age appropriate vaccination (all required vaccines due by any age) |  |  | - Number of vaccines delivered |
|  | Timeliness |  |  |  |
|  | - Receipt of vaccine/s within recommended timeframe for each vaccine | - Timeliness / on-time vaccination |  |  |
|  | - Time to vaccination (number of days spent not up to date) |  |  | - Time spent undervaccinated |
|  | - Probability of reaching one year without each immunisation |  |  |  |
|  | Appointment attendance |  |  |  |
|  | - Number of appointments attended / scheduled |  |  | - Attendance at appointments or health facilities |
|  | - Timeliness of well-child visits |  |  |  |
|  | - Attendance at >x number of appointments in a period |  |  |  |
|  | - Number of appointments scheduled or attended per child |  |  |  |
|  | - Number of children attending health centre in a certain period |  |  |  |
|  | - Kept appointment rate |  |  |  |
|  | Missed opportunities |  |  |  |
|  | - Rate of missed opportunities |  |  | - Missed/captured opportunities to discuss, plan or deliver vaccination |
|  | - Rate of captured opportunities (number of eligible visits where vaccine was administered) |  |  |  |
|  | - Rate of vaccination orders for inpatients |  |  |  |
| **COST AND COST-EFFECTIVENESS** | Cost or cost-effectiveness |  |  |  |
|  | - Of the intervention |  |  | - Cost or resource use of intervention |
|  |  |  |  | - Cost-effectiveness of interventions |
|  | - Time required to deliver the intervention | - Time taken to deliver/receive intervention |  |  |

**S4 Table B: New outcomes (not measured in trials) raised by focus group participants**

| **Outcome categories** | **New outcomes raised by BOTH parents and professionals** | **New outcomes raised by PARENTS only** | **New outcomes raised by PROFESSIONALS only** |
| --- | --- | --- | --- |
| **HEALTH OR WELL-BEING** |  |  | - Unintended impacts of interventions |
|  |  |  | - Deaths from vaccine-preventable diseases |
| **ATTITUDES OR BELIEFS** | - Attitudes or concerns about reactions or side effects |  |  |
|  | - Cultural appropriateness of intervention |  |  |
|  | - Confidence in ability to stay on schedule (behavioural control/self-efficacy) |  |  |
|  | - Confidence in communicator/provider's skills and knowledge |  |  |
|  | - Satisfaction with intervention topic and content |  |  |
|  | - Satisfaction with quantity of information |  |  |
|  | - Satisfaction with intervention delivery |  |  |
|  | - Trust in communicator/provider |  |  |
|  | - Perceived support given by communicator/ provider |  |  |
|  |  | - Attitudes or concerns about diseases | - Vaccine acceptance |
|  |  | - Attitudes or concerns about pain of vaccine delivery | - Vaccine hesitancy |
|  |  | - Confidence in efficacy or safety of vaccinations | - Political acceptability of intervention |
|  |  | - Perceived quality of intervention content | - Provider confidence in their own communication skills |
|  |  | - Perceived accuracy of intervention content | - Provider confidence in their own knowledge |
|  |  | - Perceived knowledge of communicator/provider | - Provider communication skills self-efficacy |
|  |  | - Confidence in ability to find or understand information |  |
|  |  | - Confidence in ability to judge information quality |  |
|  |  | - Satisfaction with intervention format |  |
| **DECISION-MAKING** | - Perceived control over decision-making process |  |  |
|  | - Clarity of values |  |  |
|  | - Amount and appropriateness of decision support received |  |  |
|  | - Degree of involvement in the decision-making process |  |  |
|  |  | - Confidence in decision-making ability | - Provider confidence in ability to participate in shared decision-making |
|  |  | - Confidence in planned decision | - Anticipated regret |
|  |  | - Perceived influence of intervention on decision taken | - Parents feeling important or valued in a communication encounter |
|  |  | - Satisfaction with decision support | - Shared decision-making |
|  |  |  | - Regret with decision made |
|  |  |  | - Confidence in decision made |
|  |  |  | - Parents feeling proactive or like they've taken a positive step |
| **KNOWLEDGE OR UNDER-STANDING** | - Knowledge about judging information quality |  |  |
|  | - Knowledge about where and how to find relevant information |  |  |
|  |  | - Level of information seeking or avoidance | - Community awareness about available vaccination services and organisations |
|  |  |  | - Provider knowledge about how to find additional information |
|  |  |  | - Provider knowledge about communication issues |
|  |  |  | - Being up-to-date with knowledge |
| **VACCINATION UPTAKE OR BEHAVIOURS** | - Continuity of provider |  |  |
|  |  | - Perceived competence of communicator/provider |  |
|  |  |  | - Vaccination consent card return rate |
|  |  |  | - Source of vaccination information |
|  |  |  | - Impact or reach of intervention |
|  |  |  | - Vaccination exemption or objection rate |
| **OTHER** |  |  | - Functionality of community vaccination organisations |
|  |  |  | - Whether plans are implemented as intended |
|  |  |  | - Number of house visits by community health workers |
|  |  |  | - Number of meetings of community organisations |
|  |  |  | - Number visits to health facilities by ward committees |
|  |  |  | - Parents feeling supported by their community |
|  |  |  | - Degree of outreach or engagement in intervention design and delivery |
